# Myelin-associated glycoprotein-related neuropathy associated with psoriasis: a case report

**DOI:** 10.1186/1752-1947-7-4

**Published:** 2013-01-03

**Authors:** Ken-ya Murata, Hideto Miwa, Tomoyoshi Kondo

**Affiliations:** 1 Department of Neurology, Wakayama Medical University, 811-1 Kimiidera, Wakayama 641-8510, Japan

## Abstract

**Introduction:**

Psoriasis vulgaris is a common inflammatory disease of the skin, and myelin-associated glycoprotein-related neuropathy is a chronic sensory-predominant polyneuropathy. Although both of these diseases are considered autoimmune diseases, psoriasis with concomitant myelin-associated glycoprotein-related neuropathy is very rare. Here, we report a case of myelin-associated glycoprotein-related neuropathy associated with psoriasis.

**Case presentation:**

A 66-year-old Japanese man, having experienced sternocostoclavicular pain for ten years, was admitted to our hospital because of gait disturbance and numbness of the limbs. Our patient had normal cranial nerve function and normal limb muscle strength. His vibratory and position sense was severely impaired and his touch, temperature and pinprick sensations were mildly disturbed in a glove and stocking distribution. A myelin-associated glycoprotein western blot analysis showed the presence of a 91 to 94kDa band using purified human myelin-associated glycoprotein antigen. His skin lesions were moderately pruritic and Auspitz’s sign was positive. Our patient also showed osteitis of his clavicle and manubrium. We diagnosed our patient with myelin-associated glycoprotein-related neuropathy associated with psoriatic arthritis. Five days after intravenous immunoglobulin therapy, his deep sensory impairment began to improve and his sternocostoclavicular pain diminished dramatically.

**Conclusions:**

Because myelin-associated glycoprotein-related neuropathy and psoriatic arthritis are both considered autoimmune diseases, we conclude that intravenous immunoglobulin therapy is very effective for patients with an association of these diseases.

## Introduction

Psoriasis vulgaris is a common inflammatory disease of the skin characterized by erythematous, dry, scaling plaques of various sizes [1]. Myelin-associated glycoprotein (MAG)-related neuropathy is a chronic sensory-predominant polyneuropathy with less than severe motor involvement. Although both of these diseases are considered autoimmune diseases, psoriasis with concomitant MAG-related neuropathy is very rare.

## Case presentation

**Figure 1:**
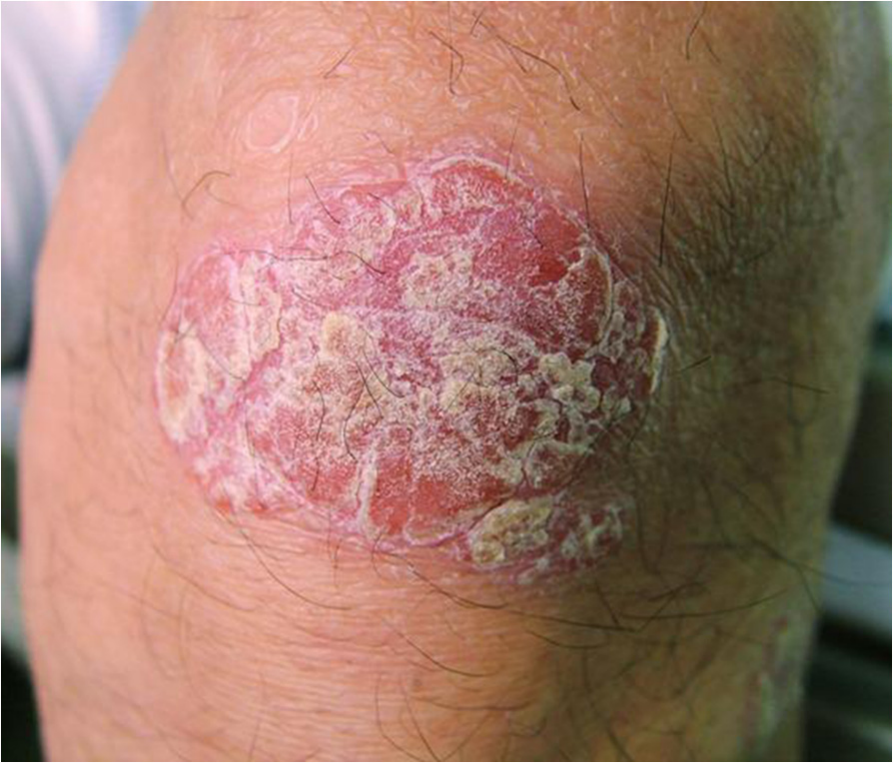
Psoriatic lesion on the knee.

A 66-year-old Japanese man, having experienced sternocostoclavicular pain for 10 years, was admitted to our hospital because of gait disturbance and numbness of the limbs. A physical examination revealed no abnormalities except for skin eruptions on the bilateral elbows, knees and hips (Figure 1). The diameter of each plaque was approximately 2cm to 5cm. These eruptions developed two years before admission and appeared as erythematous plaques covered by a silvery scaling. The skin lesions were moderately pruritic and Auspitz’s sign was positive. These findings are compatible with psoriasis vulgaris.

On neurological examination, our patient had normal cranial nerve function and normal limb muscle strength. All deep tendon reflexes were absent. His vibratory and position sense was severely impaired up to his knees. Touch, temperature and pinprick sensations were mildly disturbed in a glove and stocking distribution. His coordination was clumsy in his lower limbs because of sensory ataxia. He had gait disturbance with Romberg’s sign.

Routine blood laboratory tests were normal. His serum concentration of immunoglobulin (Ig) M was 320mg/dL (normal range: 51 to 260mg/dL), but other immunoglobulins were within the normal range. Serum immunoelectrophoresis showed IgM K-type monoclonal gammopathy. An enzyme-linked immunosorbent assay confirmed that the serum obtained from our patient before treatment contained extremely high titers of IgM antibody against MAG and sulfated glucuronyl paragloboside. A MAG western blot analysis showed the presence of a 91 to 94kDa band using purified human MAG antigen. No tumor cell proliferation was observed in a bone marrow aspiration study. Autoantibodies, including anti-deoxyribonucleic acid (DNA), anti-SS-A and anti-SS-B were not detected. Assays for ganglioside antibodies against GM1, GM2, GM3, GD1a, GD1b, GD3, GT1b, GQ1b, GA1 and Gal-C were negative. The titer of cold agglutinin was not increased. There was no cerebral spinal fluid pleocytosis, but his cerebral spinal fluid protein level was 300mg/dL.

**Figure 2:**
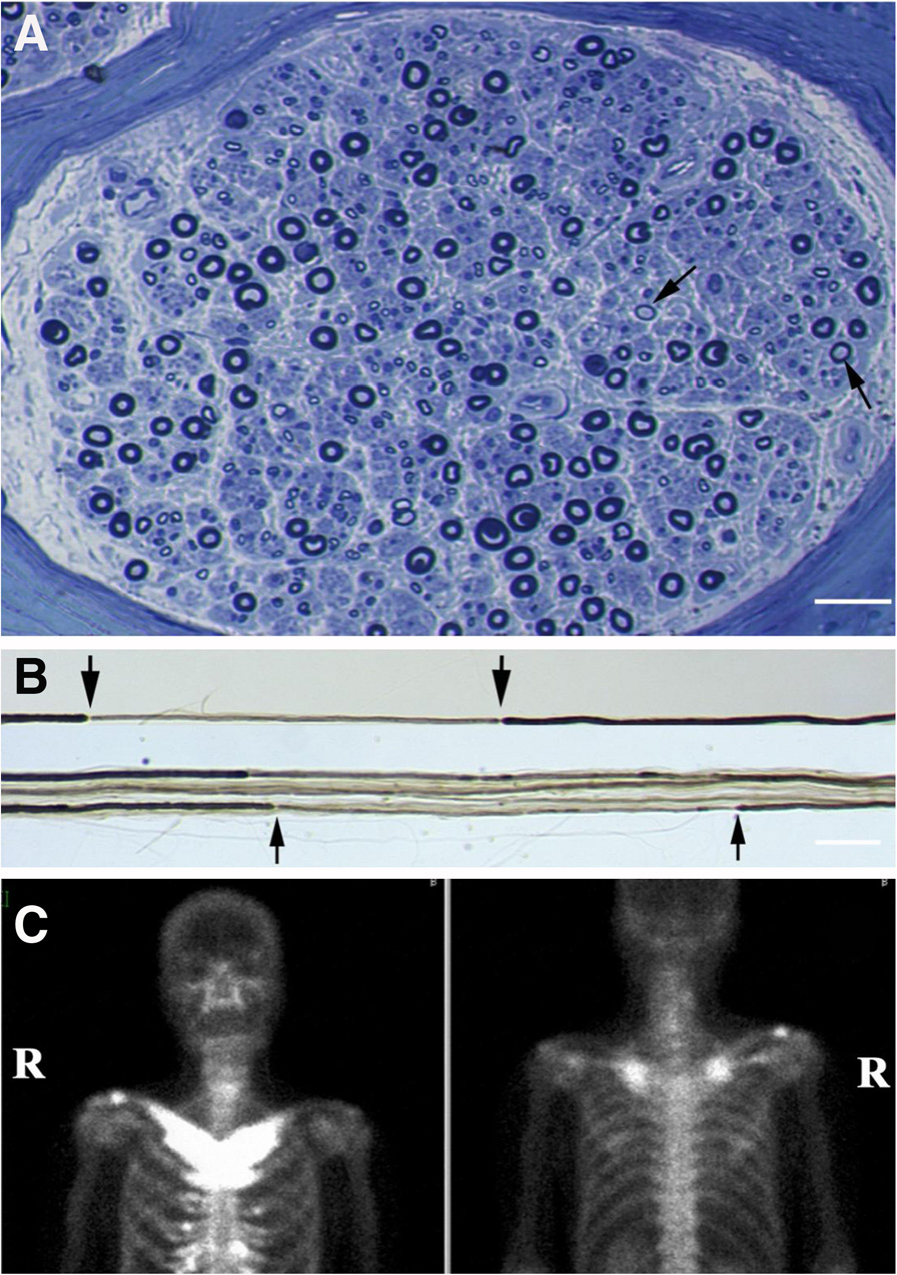
**Light micrograph of a sural nerve biopsy and a bone scintigram. **Light micrograph of a sural nerve biopsy showing a moderate reduction in the density of large myelinated fibers, with (**A**) abnormally thin myelin relative to axonal caliber (arrows) in a cross section (bar=30μm) and (**B**) teased fibers with short, thinly myelinated internodes (arrows), indicating remyelination (bar=100μm). (**C**) A bone scintigram showing an abnormal accumulation of ^99m^Tc in both of the sternocostoclavicular joints and the right clavicular region.

Motor conduction studies showed a reduced velocity in his ulnar nerve (30m/s; normal range, >50m/s) and no compound muscle action potential from his extensor digitorum brevis muscle after peroneal nerve stimulation. Sensory nerve action potentials could not be detected in either upper or lower limbs. A sural nerve biopsy showed a moderate reduction in density of large myelinated fibers (Figure 2A), with remyelinated fibers comprising 21.8% of all teased fibers (Figure 2B). His sternocostoclavicular joints and right clavicular region showed active uptake of the radioisotope ^99m^Tc, consistent with osteitis of the clavicle and manubrium (Figure 2C).

Our patient was human leukocyte antigen B39 positive, but negative for B27. We diagnosed our patient with MAG-related neuropathy associated with psoriatic arthritis. Intravenous immunoglobulin (IVIg) therapy, 400mg/kg/day for five days, was administered. His deep sensory impairment began to improve five days after starting IVIg therapy. The severity of his sensory ataxia was evaluated by the size of the estimated area when our patient stood on a stabilometer with closed stance under open eye or closed eye conditions. The estimated areas under open eye conditions showed no remarkable changes after IVIg treatment (pretreatment area, 10.0cm^2^; post-treatment area, 10.5cm^2^). However, estimated areas under closed eye conditions became smaller after IVIg treatment (pretreatment area, 56.2cm^2^; post-treatment area, 37.4cm^2^). One week later, our patient observed that his sternocostoclavicular pain was dramatically diminished and his inflammatory markers improved (erythrocyte sedimentation rate decreased from 66 to 20mm in the first hour and C-reactive protein decreased from 3.63 to 1.28mg/dL). The improvement of his sensory ataxia persisted for at least three months.

## Discussion

Our patient had psoriasis and MAG-related neuropathy at the same time. The selection of an effective immunosuppressive therapy for both diseases is very difficult, because MAG-related neuropathy is related to B cell immunity, whereas psoriasis is considered an autoimmune disease with T cells playing a key role in the pathogenesis [1]. Indeed, IVIg therapy is effective for patients with chronic inflammatory demyelinating polyneuropathy, but it is not a standard therapy for a patient with MAG-related neuropathy. The small clinical trials that have been reported show no convincing effect, other than short term [2]. Although the combined administration of IVIg and interferon-β-1a is effective in patients with acute inflammatory demyelinating polyneuropathy [3], it is not obvious that this combined therapy is effective in patients with MAG-related neuropathy.

Several therapies have been proposed for psoriasis, including topical corticosteroid, vitamin D analogues, phototherapy and systemic immunosuppressants. Tumor necrosis factor alpha (TNF-α) is an important immunomodulator that plays a role in immune system development, immune response regulation and T-cell-mediated tissue injury [4]. Recently, TNF-α blockers were suggested for the treatment of inflammatory disorders including rheumatoid arthritis, psoriasis and psoriatic arthritis [5]. However, they can induce autoimmunity and have induced peripheral nerve demyelinating diseases such as chronic inflammatory demyelinating polyneuropathy [6]. Although there was no significant improvement in the cutaneous lesions of psoriasis, IVIg therapy is known to be effective for psoriasis or psoriatic arthritis [7,8]. Therefore, we chose IVIg therapy because the symptoms of MAG-related neuropathy may be exacerbated by anti-TNF-α therapy. Fortunately, IVIg therapy dramatically improved not only the sensory ataxia but also the sternocostoclavicular pain.

There are a few publications that describe patients with psoriasis who develop polyneuropathy, and most of these patients showed axonopathy [9-11]. Demyelinating neuropathy associated with psoriasis is very rare, and MAG-related neuropathy is classified as a demyelinating neuropathy [2]. Thus, concomitant occurrence of psoriasis and MAG-related neuropathy may be by chance. Psoriasis is easy to observe, but MAG-related neuropathy is not. Therefore, close attention should be paid to the coexistence of MAG-related neuropathy with psoriasis to avoid side effects of immune therapy, especially anti-TNF-α therapy.

## Conclusion

We conclude that IVIg therapy is very effective for patients with MAG-related neuropathy associated with psoriatic arthritis.

## Consent

Written informed consent was obtained from the patient for publication of this case report and any accompanying images. A copy of the written consent is available for review by the Editor-in-Chief of this journal.

## Competing interests

The authors declare that they do not have any competing interests.

## Authors’ contributions

KM was a major contributor in writing the manuscript. HM and TK participated in the intellectual content, the analysis of data and the writing of the manuscript and take public responsibility for it. HM and TK reviewed the manuscript. All authors read and approved the final version of the manuscript.
